# Suicidal behavior in a migrant majority population and impact on trends during the early Covid-19 period: a cross sectional study in Qatar

**DOI:** 10.1007/s44202-022-00040-8

**Published:** 2022-05-25

**Authors:** Majid AlAbdulla, Shuja Mohd Reagu, Mohamed H. M. O. Hassan, Nahid M. Elhassan, Sagda Sayed, Ibrahim Makki, Marwa Elzain, Ovais Wadoo, Rajeev Kumar

**Affiliations:** 1 Department of Psychiatry, Hamad Medical Corporation, Doha, Qatar; 2 College of Medicine, Qatar University, Doha, Qatar; 3 Weill Cornell Medicine—Qatar, Doha, Qatar; 4 Mental Health Services, Hamad Medical Corporation, P.O. Box 3050, Doha, Qatar

**Keywords:** Suicidal behaviors, Qatar, EMR (Eastern Mediterranean Region) region, Migrant mental health

## Abstract

**Background:**

Qatar is a high-income country with 90% of the population being economic migrants from low income countries. Due to this unique population composition, it has been suggested that Qatar may not follow suicide trends of high-income countries. Additionally, there is paucity of information on suicidal trends and rates due to social and cultural reasons. The Covid-19 pandemic has additionally impacted mental health of migrants differently form native Qataris.

**Objectives:**

This study explores suicidal behavior trends among individuals attending the main Emergency Department in the state of Qatar for mental health emergencies. The study also compared these trends for pre-pandemic period to early post pandemic period.

**Methods:**

A cross-sectional study of individuals attending the main emergency department of the country from 1st December 2019 to 30th June 2020 was carried out using a composite data collection form. This identified 799 individuals presenting with mental health emergencies. Suicidal behaviors, relevant sociodemographic data, along with factors known to be associated with suicidal behaviors were collected for this group.

**Results:**

24.9% (n = 199) of the sample presented with suicidal behaviors. Younger age (31.16 ± 9.497), current hopelessness (70; 54.7%), history of suicidal thoughts (50; 47.2%), history of suicidal attempts (43; 34.7%), history of self-harm thoughts (35; 39.3%), history of self-harm attempts (41; 37.6%) were highly significantly associated with suicidal behavior (*p* < 0.01). Qataris formed 27% of the group presenting with suicidal behaviors although they constitute only around 10% of the population. There was no significant change in the rate of presenting with suicidal behaviors during the early stages of the Covid-19 pandemic.

**Conclusions:**

This study reports an annual incidence of suicidal behaviors in Qatar lower than that has been previously reported. The authors surmise that this may be due to improved availability and early intervention of mental health services and decreased stigma around mental health.

**Supplementary Information:**

The online version contains supplementary material available at 10.1007/s44202-022-00040-8.

## Introduction and background

WHO (World Health Organistaion) has classified suicide as serious global health concern that results in over 800,000 deaths per year [1]. The WHO designated Eastern Mediterranean Region (EMR), a region with geographical and cultural similarities, which extends from Afghanistan to Morocco reports suicide as the 25th leading cause of death with the Muslim majority countries in this region reporting lowest suicide rates in the world [2]. It has been claimed that strong religious affiliations and identity can be protective factors against suicide [3–5]. However, these claims have to be posited against concerns around paucity of robust suicidal behavior data from the Islamic countries in this region [2, 6, 7]. It has been suggested that criminalizing suicide leads to underreporting in these countries [8, 9]. Additionally, cultural and religious stigma against suicide further relates to underreporting of rates [6, 10]. The picture is further complicated by a lack of national, state held national registers for suicide and the published rates of suicide come from studies by independent researchers who may not have a comprehensive access to all sources of data [2, 3].

Moreover, some of the countries in the EMR like Qatar, Kuwait and UAE have experienced very rapid economic growth in recent past and are classified as high-income countries as opposed to the rest of the countries in the region [11]. Although WHO notes that 79% of the suicides occur in low to middle income countries, these three high income countries are unique in that their rapid economic growth has significantly impacted the composition of their resident populations [12, 13] thereby making the picture more complicated. In fact, boom in economy and infrastructure development has led to an influx of economic immigrants mostly form low income countries like India, Bangladesh and Nepal which have much higher reported incidences of suicide than the EMR region [14–16].

Concerns have been raised around the working conditions and mental health wellbeing of these immigrants, including the number of suicides amongst them, in these countries [17–19].

Previous studies on suicidal and parasuicidal behaviors in Qatar reported that the rates were higher among the immigrant workers form the eastern Asian nations and this was replicated in studies in Dubai and Kuwait [7, 20–22]. However, most of these studies present data that are over a decade old including the only one from Qatar. Considering that this region has been experiencing rapid changes in both demographic and healthcare profiles [23–25], there is real paucity of up to date information on suicidal behavior among residents in Qatar.

Moreover, this higher rate amongst the migrant population groups has also been attributed to separation from families and deprivation [17, 26]. Incidentally, the contact with families has been significantly impacted by the restriction of movement due to the global Covid-19 pandemic. The impact of the pandemic on mental health has been well documented in general and the impact on travel back to home countries on immigrants residing in Qatar in particular [27, 28]. Previous global epidemics have shown associations between worsening mental health and increased suicidality, for instance in the USA during the 1918–19 influenza pandemic [29] and in Hong Kong during the 2003 severe acute respiratory syndrome (SARS) epidemic [30].

Against this background and in context of the unique sociodemographic characteristics of the population in Qatar, an exploration of suicidal behavior rates was carried out. This study was additionally designed to explore whether the additional burden of limitations on travel and social contact due to the Covid-19 pandemic has had a further impact on suicidal behavior of Qatari residents including on economic immigrants. This makes this study a first of its kind in Qatar and possibly the wider region in that it explores suicidal behaviors within the context of the Covid-19 pandemic.

## Method

### Data source and study design

Our study was an observational, cross-sectional design which extracted retrospective data from Electronic Patient Records (EPR) at the Emergency Department (ED) of the main state-run Hospital, Hamad General Hospital, the major provider of healthcare in Qatar, over the identified period. In this report, we present data that we gathered from 1 December 2019 to 30 June 2020. In Qatar, the cases of COVID-19 started to emerge from late February 2020 and started to peak by June and July [31]. This allowed us to compare data from December 2019 to end of February 2020 (pre-COVID-19) with data from March through June 2020 (Post-COVID-19). Ethical approval was sought and received from The Institutional Review Board of the Hamad Medical Corporation (MRC-05-095).

### Participants

We included all first presentations during the identified period of those people presenting with “suicidal behavior”—which was defined as thoughts of suicide (an intent to die), thoughts of deliberate self-harm (no definite intent to die), attempted suicide, and attempted deliberate self-harm to the ED of our main public hospital. We included only new presentations each month, and if those were presented again the following month, those were excluded, as we wanted to see if there has been an increase of new cases following the COVID-19 pandemic. Qatar had a population of about 2.88 million at the time of this study out of which around 89% are expatriates from several countries, and the rest are native Qataris [32]. The ED of the main state-run hospital in Qatar is based in the capital Doha and serves the capital and its suburbs which accounts for over 90% of the population of Qatar. This main ED receives around 3000 new visits each day. The two other state-run Emergency departments are relatively smaller and the private institutions in the country do not have any significant emergency psychiatric facilities. Every visit which mentioned suicidal behavior, as described above, during this period was included in this study. The only exclusion was if the individuals were under 18 years of age.

### Measures

We developed a composite data collection form. The first section included demographic characteristics, COVID-19 status, associated physical comorbidities, smoking status, alcohol and substance use history, and past psychiatric history. The second section included information about symptoms that are known to be associated with suicidal behaviors like presence of current mental disorders, documented evidence of hopelessness, worthlessness, acute stressors, followed by a detailed account of current suicidal behavior, past suicidal behavior, and evidence of social isolation/entrapment/quarantine during the presentation. Initial draft of the questionnaire was piloted on 30 EPR by three researchers to assess feasibility of the questionnaire. Modifications were made to the questionnaire to account for missing or unclear information and a final version was approved after discussion with the wider team. To achieve maximum reliability among the raters, two training sessions about the rating methods and terms were carried out. As a final step, 60 of the modified questionnaires were completed again by the three researchers and we achieved high interrater reliability.

### Patient and public involvement

Patients or the public were not involved in the design or conduct of this research or in the dissemination of the research plans.

### Data analyses

We analyzed the data using SPSS version 26 software. Initially, we conducted a simple frequency analysis of those presented each month with suicidal behavior using the total presentations as the denominator. We then conducted a univariate analysis comparing those with suicidal behavior and those without using *t*-tests and chi-square (χ^2^) tests for comparisons of continuous variables and categorical variables, respectively. Furthermore, we used a binary logistic regression to examine the potential risk factors predicting suicidal behavior, using the enter method after removing variables that had multicollinearity.

## Results

From 1 December 2019 to 30 June 2020 (seven months), a total of 799 individuals with mental health issues presented to the ED, which comprised the total sample. Out of these, 199 (24.9%) presented with suicidal behavior. 524 (65.6%) of this sample were males and 275 (34.4%) females. The mean age of this sample was 35.44 ± 12.60 years. Most of the individuals of the total sample were living with family (437; 54.7%), Islam was the cited religion for 357 (44.7%), and a third of the total sample was Qataris (241; 30.2%). More demographic characteristics are shown in Table 1.

**Table 1:** Demographic characteristics of the study sample (N = 799)

Characteristics	n (%) or Mean ± SD
Age in years	35.44 ± 12.60
Gender	
Male	524 (65.6)
Female	275 (34.4)
Marital status	
Single	255 (31.9)
Married	335 (41.9)
Divorced	34 (4.3)
Separated	7 (0.9)
Widowed	5 (0.6)
Unknown	163 (20.4)
Employment	
Yes	387 (48.4)
No	287 (35.9)
Unknown	125 (15.6)
Living status	
Living alone	30 (3.8)
With a sharing group	209 (26.2)
With family	437 (54.7)
Unknown	123 (15.4)
Religion	
Islam	357 (44.7)
Hindu	15 (1.9)
Christian	5 (0.6)
Unknown	404 (50.6)
Nationality	
Qatari	241 (30.2)
Asian	287 (35.9)
Middle Eastern (excluding Qatar)	115 (14.4
African	87 (10.9)
European	16 (2.0)
North American	8 (1.0)
Others	19 (2.4)
Unknown	26 (2.0)
Residency	
Citizens	243 (30.4)
Residents	479 (59.9)
Visitors	36 (4.5)
Unknown	41 (5.1)

The clinical characteristics are shown in Table 2. Of note, 194 (24%) were current smokers, 112 (14%) were current alcohol consumers, 84 (10.5%) were current substance users. Only 23 (2.9%) were COVID-19 positive.

**Table 2:** Clinical characteristics of the study sample (N = 799)

Characteristics	n (%)
Suicidal behavior (cases)	199 (24.9)
Non-suicidal acute cases (controls)	600 (75.1)
Current smoking	
Yes	194 (24.3)
No	313 (39.2)
Unknown	292 (36.5)
Current alcohol use	
Yes	112 (14.0)
No	450 (56.3)
Unknown	237 (29.7)
Current substance use	
Yes	84 (10.5)
No	473 (59.2)
Unknown	242 (30.3)
Past psychiatric history	
Yes	460 (57.6)
No	295 (36.9)
Unknown	44 (5.5)
Known to our mental health service	
Yes	301 (37.7)
No	498 (62.3)
COVID-19 status	
Positive	23 (2.9)
Negative	442 (55.3)
Unknown or not tested	334 (41.8)
Current hopelessness documented	
Yes	90 (11.3)
No	316 (39.5)
Unknown	393 (49.2)
Current psychiatric diagnosis (broad category)	
Psychosis	198 (24.8)
Mood disorders	261 (32.7)
Anxiety disorders	38 (4.8)
Personality disorders	42 (5.3)
Alcohol and substance use disorders	44 (5.5)
Others	126 (15.8)
No psychiatric diagnosis	53 (6.6)
Unknown (not available)	37 (4.6)

Suicidal-behavior related characteristics are shown in Table 3. Those with thoughts of suicide were 137 (17.1%), thoughts of self-harm 46 (5.8%), attempted suicide 83 (10.4%), and attempted self-harm 64 (8%). The most common methods of thoughts or attempts were overdose (42; 5.3%), slashing or cutting (37; 4.6%), jumping from heights (19; 2.4%), and hanging (16; 2%). History of suicidal thoughts/self-harm thoughts was present in 127 (15.9%), and history of attempts was present in 121 (15.2%) cases. Acute stressors were present in a substantial number of cases (457; 57.2%).

**Table 3:** Suicidal behavior-related characteristics of the sample (N = 799)

Characteristics	n (%)
Thoughts of suicide	
Yes	137 (17.1)
No	588 (73.6)
Unknown	74 (9.3)
Thoughts of self-harm	
Yes	46 (5.8)
No	549 (68.7)
Unknown	204 (25.5)
Attempted suicide	
Yes	83 (10.4)
No	550 (68.8)
Unknown	166 (20.8)
Attempted self-harm	
Yes	64 (8.00
No	511 (64.0)
Unknown	224 (28.0)
Types of suicidal behavior (thoughts or attempts)	
Overdose	42 (5.3)
Slashing/cutting	37 (94.6)
Jumping from heights	19 (2.4)
Hanging	16 (2.0)
Hitting self	6 (0.8)
Stabbing self	6 (0.8)
Others	34 (4.2)
Not applicable or unknown	634 (79.3)
Past history of suicidal thoughts/self-harm thoughts	
Yes	127 (15.9)
No	203 (25.4)
Unknown	514 (64.3)
Past history of suicidal attempts/self-harm-attempts	
Yes	121 (15.2)
No	267 (33.4)
Unknown	466 (58.3)
Acute stressors	
Yes	457 (57.2)
No	100 (12.5)
Unknown	242 (30.4)

### Time trends in suicidal behavior

**Fig. 1:**
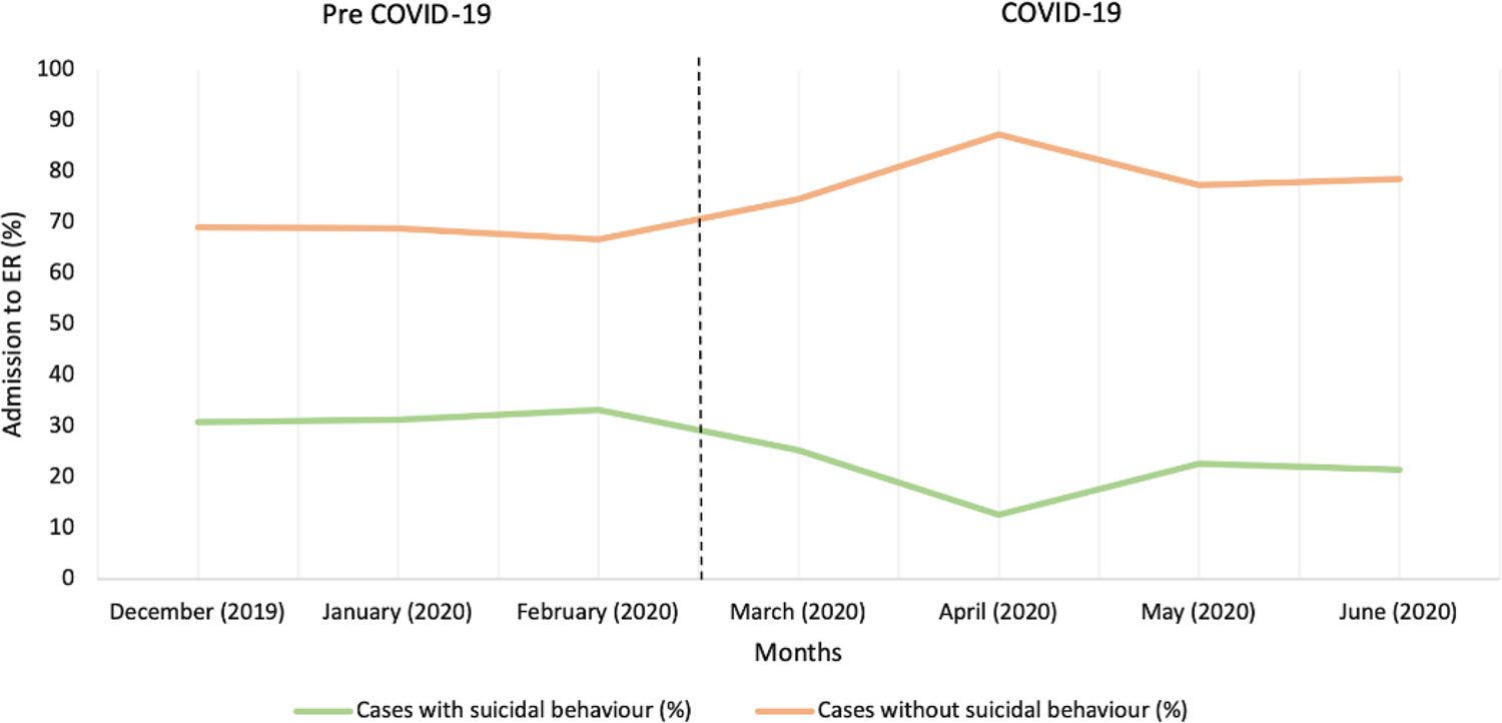
Trend (%) of those with suicidal behavior and those without (N = 799). *ER* emergency room

Month-by-month frequency of those with suicidal behavior and those without are shown in Fig. 1. In December 2019, there were 29 (30.9%), in January 2020, 30 (31.3%), February 17 (33.3%), March 44 (25.4%), April 8 (12.7%), May 34 (22.7%), and in June 37 (21.5%) of cases with suicidal behavior, showing no increase in trend. Rather there was a significant drop in April. However, the acute cases without suicidal behavior showed a steady increase from March 2020, peaking in April and then coming down by June.

Univariate analysis comparing those with suicidal behavior and those without are shown in Table 4. Younger age (31.16 ± 9.497), being non-married (91; 54.2%), currently smoking (71; 52.6%), using alcohol (43; 28.7%) were highly significantly associated with suicidal behavior compared to those without (p < 0.01). Similarly, experiencing acute psychosocial stress (156; 91.2%), current hopelessness (70; 54.7%), and having a history of suicidal thoughts (50; 47.2%), suicidal attempts (43; 34.7%), self-harm thoughts (35; 39.3%), or self-harm attempts (41; 37.6%) were highly significantly associated with suicidal behavior compared to those without (*p* < 0.01).

**Table 4:** Univariate analysis comparing those with suicidal behavior and those without (N = 799)

Characteristic	Clinical diagnosis^a^ n (%) or mean ± SD		*P* value (2-tailed)
	Cases with suicidal behavior	Cases without suicidal behavior	
Age	31.16 ± 9.50	36.85 ± 13.18	0.000
Gender			
Male	125 (62.8%)	399 (66.5%)	0.343
Female	74 (37.2%)	201 (33.5%)	
Ethnicity			
Qatari	65 (32.7%)	176 (29.3%)	0.375
Non-Qatari	134 (67.3%)	424 (70.7%)	
Marital status			
Married	77 (45.8%)	258 (55.1%)	0.038
Non-married (all except unknown)	91 (54.2%)	210 (44.9%)	
Employment status			
Employed	101 (54.9%)	286 (58.4%)	0.416
Not employed	83 (45.1%)	204 (41.6%)	
Living status			
Living with family	102 (60%)	335 (66.2%)	0.143
Living alone or in group	68 (40%)	171 (33.8%)	
Religion			
Islam	92 (92%)	265 (89.8%)	0.525
Non-Islam	8 (8%)	30 (10.2%)	
Current smoking			
Yes	71 (52.6%)	123 (33.1%)	0.000
No	64 (47.4%)	249 (66.9%)	
Current alcohol use			
Yes	43 (28.7%)	69 (16.7%)	0.002
No	107 (71.3%)	343 (83.3%)	
Current substance use			
Yes	32 (20.1%)	52 (13.1%)	0.035
No	127 (79.9%)	346 (86.9%)	
Past psychiatric history			
Yes	106 (54.6%)	354 (63.1%)	0.037
No	88 (45.4%)	207 (36.9%)	
Recent acute psychosocial stress			
Yes	156 (91.2%)	301 (78.0%)	0.000
No	15 (8.8%)	85 (22.0%)	
COVID-19 positive status			
Yes	6 (5.4%)	17 (4.8%)	0.818
No	106 (94.6%)	336 (95.2%)	
Current hopelessness			
Yes	70 (54.7%)	20 (7.2%)	0.000
No	58 (45.3%)	258 (92.8%)	
Past history of suicidal thoughts			
Yes	50 (47.2%)	32 (17.9%)	0.000
No	56 (52.8%)	147 (82.1%)	
Past history of suicidal attempts			
Yes	43 (34.7%)	23 (11.0%)	0.000
No	81 (65.3%)	186 (89.0%)	
Past history of self-harm thoughts			
Yes	35 (39.3%)	10 (6.3%)	0.000
No	54 (60.7%)	148 (93.7%)	
Past history of self-harm attempts			
Yes	41 (37.6%)	14 (7.5%)	0.000
No	68 (62.4%)	173 (92.5%)	
Quarantined			
Yes	8 (5.9%)	25 (5.9%)	0.981
No	128 (94.1%)	396 (94.1%)	

^a^Percentages in parentheses are relative to valid total cases of the respective column (i.e., missing values are excluded from the denominator)

The final regression model showed that having thoughts of hopelessness was a significant risk factor in predicting suicidal behavior (Table 5).

**Table 5:** Logistic regression analysis for predictors of those who had suicidal behavior compared those without (N = 799)

Variable	β	SE	Wald	OR	95% CI for OR
Age in years	− 0.012	0.032	0.137	0.988	0.927–1.053
Marital status	− 1.069	0.715	2.234	0.343	0.084–1.395
Current smoking status	0.799	0.701	1.297	2.222	0.562–8.782
Current alcohol use	0.719	0.854	0.708	2.051	0.385–10.936
Current substance use	0.267	0.927	0.083	1.306	0.212–8.034
Past psychiatric history	− 1.211	0.677	3.204	0.298	0.079–1.122
Recent psychosocial stress	0.758	0.724	1.098	2.135	0.517–8.821
Current hopelessness	3.368	0.916	13.532	29.016*	4.823–174.550
Past history of deliberate self-harm attempts	1.010	0.888	1.294	2.746	0.482–15.649
Past history of suicidal attempts	1.587	0.920	2.971	4.887	0.805–29.683

Variables past history of suicidal thoughts, and deliberate self-harm thoughts were removed due to multicollinearity. Cox and Snell R^2^ = 0.450, Nagelkerke R^2^ = 0.621, Hosmer and Lemeshow goodness-of-fit χ^2^ = 3.942, *p* = 0.862

^*^p =  < 0.001

## Discussion

### Suicidal behavior trends; general epidemiological observations

This study set out to explore presentations to emergency care with suicidal behaviors in Qatar and also whether the Covid-19 pandemic had any impact on these presentations. The main finding of this study, which is a first in almost a decade, was that almost a quarter (24.9%) of the psychiatric presentations to Emergency Department presented with some form of suicidal behaviors. This gave the annual incidence of the suicidal behaviors during this study period, which included the pre-pandemic period, to be at 14.2 per 100,000 of the adult population. Qatari natives formed 27% of the group presenting with suicidal behaviors although they constitute only around 10% of the population. The majority of the manual laborers are from the Indian subcontinent and they constituted 23.3% of the suicidal behavior group. The Asian community comprises around 56% of the population in Qatar [32].

Due to its unique demographic make-up, males in Qatar outnumber females in the ratio of 3:1 [32]. The annual incidence of suicidal behavior for males was at 11.7 per 100,000 adult males and for females, it was significantly higher at 20.8 per 100,000 adult females, (z statistic—5.13 and p < 0.00001).

Overall, these figures are much lower than the only other study [20] exploring the suicidal behaviors presenting to the Emergency Department of the same hospital in Qatar which reported on data that was collected around 10 years ago. There have been no changes in the legal status to reporting suicide since the previous study. However, the population composition and the mental health services in Qatar have undergone significant changes since that time [32–34]. The regular mental health services are provided by the state heavily subsidized and free of charge in emergency to all residents irrespective of the nationality status. Additionally, National Mental Health Strategy launched in 2013 focused on promotion and prevention of mental ill health through service integration [33]. We believe that the national focus on early detection and prevention through mental health promotion has led to improvement in service delivery, development of integrated community mental health services, recruitment of experts has contributed to this apparent improvement in suicidal behavior attendance in the emergency department [34]. Finally, changes in social attitudes, including stigma, to mental health and treatment of mental illness have also experienced changes over the recent years in Qatar [35]. This last change may explain the relative increase in the proportion of local Qataris and individuals of ethnic Arab origin seeking help for suicidal behaviors.

### Covid-19 suicidal behavior trends

Another main finding of this study is that there was no significant change in the rate of suicidal behaviors seeking emergency care in the immediate post covid-19 pandemic period in Qatar. In fact, there was a drop in the number of cases presenting with suicidal behaviors.

This is an interesting finding given that there was an increase in number of people seeking help for mental health issues in the same period. The impact of Covid-19 on mental health has been extensively studied [36] and the increase in mental ill health rates in this study follows the trends across the globe. We hypothesize that during the early post pandemic period, the thoughts of getting infected and consequences of the emerging pandemic in a rapidly changing situation did not immediately translate into longer term hopelessness and consequently increased suicidal behaviors. This wasn’t just at the individual level but even state policies differed in their response to the pandemic and this was underlined by lack of consensus globally around the nature and severity of the pandemic [37]. It is also possible that fear of infection kept people away from attending hospital emergency departments for non-Covid 19 related issues during these early stages potentially impacting emergency psychiatry attendance rates [38, 39]. In fact, studies looking into suicidal behaviors in general population rather than emergency department attendees during the early post pandemic period reported increased rates of suicidal behaviors [40, 41]. Qatar was able to implement quick and sweeping changes in delivery of healthcare, including mental health care, to manage the pandemic and its fallout owing to its small size and default centralization of services [42]. This included rapid and easier access to mental health services through tele-psychiatry services and Covid-19 mental health helpline [43]. Taken together these might explain the relative drop in suicidal behavior cases attending the ED during the early post covid-19 lockdown period. The suicidal behavior rates in this study started picking up as the pandemic and its impact on all aspects of life stared becoming clearer and prolonged.

### Trends in the migrant population

Finally, it is interesting to note that despite the restrictions on travel and impact on livelihoods, the suicidal behaviors in the economic immigrant population did not vary significantly over this study period. This was despite this subgroup of the population in Qatar having experienced significantly more mental health issues during the pandemic and the self-reported lack of contact with their families as the main contributing factor to their mental distress [28]. Again, we surmise that ease of access to mental health help and the relatively short duration of experience of Covid-19 related restrictions and stresses Covid-19 pandemic could be the possible reasons explaining this finding.

We suspect that as our wider study continues to collect data, and with the continued restrictions of this pandemic affecting lives, we will see a change in the observed trends so far.

## Conclusion

Despite reported concerns around relatively high suicidal behavior rates in Qatar, particularly among the low paid economic immigrants, our data did not support such claims. Provision of good quality, easily accessible and equitable mental health services during and before the Covid-19 pandemic has helped manage mental ill health and suicidal behaviors among the residents of Qatar. There is a possibility that as the Covid-19 pandemic evolves and persists and its long-term impacts on all aspects of life become clearer, the rates of mental ill health and the associated suicidal behaviors may increase. We expect that mental health services will continue to see elevated rates of mental ill health as the pandemic continues to unfold and will require increased allocation of resources and attention from healthcare policy makers.

## Strengths and limitations

While the strength of this study lies in that it presents the most recent data on suicidal behaviors in Qatar during the Covid-19 pandemic, collected by trained psychiatrists however, it does have limitations.

The study relies on retrospective data collected from electronic patient records which do not always capture all the variables we were exploring. Additionally, study only presents findings from the early post pandemic period and may not report actual trends as the pandemic develops and leaves lasting impact on people’s lives.

## Supplementary Information

Below is the link to the electronic supplementary material.

Supplementary file 1 (PDF 90 KB)

## Data Availability

The datasets used and/or analysed during the current study are available from the corresponding author on reasonable request.

## References

[1] https://www.who.int/news-room/fact-sheets/detail/suicide. Accessed 15 May 2021.

[2] Morovatdar N, Moradi-Lakeh M, Malakouti SK, Nojomi M. Most common methods of suicide in Eastern Mediterranean region of WHO: a systematic review and meta-analysis. Arch Suicide Res. 2013;17(4):335–44. 10.1080/13811118.2013.801811. 24224668 10.1080/13811118.2013.801811

[3] Gearing RE, Alonzo D. Religion and suicide: new findings. J Relig Health. 2018;57(6):2478–99. 29736876 10.1007/s10943-018-0629-8

[4] Stack S, Kposowa AJ. Religion and suicide acceptability: a cross-national analysis. J Sci Study Relig. 2011;50(2):289–306. 21969937 10.1111/j.1468-5906.2011.01568.x

[5] Shah A, Chandia M. The relationship between suicide and Islam: a cross-national study. J Inj Violence Res. 2010;2(2):93. 21483204 10.5249/jivr.v2i2.60 PMC3134910

[6] Pritchard C, Amanullah S. An analysis of suicide and undetermined deaths in 17 predominantly Islamic countries contrasted with the UK. Psychol Med. 2007;37(3):421. 17176500 10.1017/S0033291706009159

[7] Dervic K, Amiri L, Niederkrotenthaler T, Yousef S, Salem MO, Voracek M, Sonneck G. Suicide rates in the national and expatriate population in Dubai, United Arab Emirates. Int J Soc Psychiatry. 2012;58(6):652–6. 22169999 10.1177/0020764011430038

[8] Lester D. Suicide and Islam. Arch Suicide Res. 2006;10(1):77–97. 16287698 10.1080/13811110500318489

[9] Khan MM, Hyder AA. Suicides in the developing world: case study from Pakistan. Suicide Life Threat Behav. 2006;36(1):76–81. 16676628 10.1521/suli.2006.36.1.76

[10] Sarfraz MA, Castle D. A Muslim suicide. Australas Psychiatry. 2002;10(1):48–50.

[11] https://www.who.int/phe/health_topics/outdoorair/databases/country_grouping_2016.pdf. Accessed 15 May 2021.

[12] https://www.who.int/news-room/fact-sheets/detail/suicide. Accessed 15 May 2021.

[13] Sater J. Migration and the marginality of citizenship in the Arab Gulf Region: human security and high modernist tendencies. In: The crisis of citizenship in the Arab world. Brill: Leiden; 2017. p. 224–45.

[14] Aggarwal S. Suicide in India. Br Med Bull. 2015. 10.1093/bmb/ldv018. 25958380 10.1093/bmb/ldv018

[15] Sharmin Salam S, Alonge O, Islam MI, Hoque DME, Wadhwaniya S, Ul Baset MK, El Arifeen S. The burden of suicide in rural Bangladesh: magnitude and risk factors. Int J Environ Res Public Health. 2017;14(9):1032. 28891939 10.3390/ijerph14091032 PMC5615569

[16] Thapaliya S, Sharma P, Upadhyaya K. Suicide and self harm in Nepal: a scoping review. Asian J Psychiatry. 2018;32:20–6. 10.1016/j.ajp.2017.11.018 29202423

[17] Al-Maskari F, Shah SM, Al-Sharhan R, Al-Haj E, Al-Kaabi K, Khonji D, Bernsen RM. Prevalence of depression and suicidal behaviors among male migrant workers in United Arab Emirates. J Immigr Minor Health. 2011;13(6):1027. 21503739 10.1007/s10903-011-9470-9

[18] Bhugra D, Gupta S, Bhui K, Craig TOM, Dogra N, Ingleby JD, Tribe R. WPA guidance on mental health and mental health care in migrants. World Psychiatry. 2011;10(1):2. 21379345 10.1002/j.2051-5545.2011.tb00002.x PMC3048516

[19] https://www.theguardian.com/global-development/2021/feb/23/revealed-migrant-worker-deaths-qatar-fifa-world-cup-2022. Accessed 15 May 2021.

[20] Al-Amin H, Singh R, Abdulrazzak M, Ghuloum S. Psychosocial and clinical profiles of the cases visiting the emergency department due to accidental self-harm and suicide attempts in Doha, Qatar: a retrospective study. Community Ment Health J. 2020. 10.1007/s10597-020-00650-3. 32506256 10.1007/s10597-020-00650-3 PMC7835152

[21] Koronfel AA. Suicide in Dubai, United Arab Emirates. J Clin Forensic Med. 2002;9(1):5–11. 15274957 10.1054/jcfm.2002.0514

[22] Al-Waheeb S, Al-Kandery N, Al-Omair N, Mahdi A. Patterns of suicide in Kuwait from 2014 to 2018. Public Health. 2020;187:1–7. 32866817 10.1016/j.puhe.2020.07.032

[23] Goodman A. The development of the Qatar healthcare system: a review of the literature. Int J Clin Med. 2015;6(03):177.

[24] Rajan SI. Demography of the gulf region. In: South Asian migration in the gulf. Cham: Palgrave Macmillan; 2018. p. 35–59.

[25] https://www.khaleejtimes.com/news/uae-health/rewind-50-years-uae-healthcare-has-come-a-long-way. Accessed 15 May 2021.

[26] Ahmed SH, Zainulabdin F. Dubai syndrome in Karachi. J Pak Med Assoc. 1991;41(1):10–2. 1900543

[27] Xiong J, Lipsitz O, Nasri F, Lui LM, Gill H, Phan L, et al. Impact of COVID-19 pandemic on mental health in the general population: a systematic review. J Affect Disord. 2020. 10.1016/j.jad.2020.08.001. 32799105 10.1016/j.jad.2020.08.001 PMC7413844

[28] Reagu S, Wadoo O, Latoo J, Nelson D, Ouanes S, Masoodi N, Alabdulla M. Psychological impact of the COVID-19 pandemic within institutional quarantine and isolation centres and its sociodemographic correlates in Qatar: a cross-sectional study. BMJ Open. 2021;11(1): e045794. 10.1136/bmjopen-2020-045794 PMC7852068 33518530

[29] Wasserman IM. The impact of epidemic, war, prohibition and media on suicide: United States, 1910–1920. Suicide Life Threat Behav. 1992;22(2):240–54. 1626335

[30] Cheung Y, Chau PH, Yip PS. A revisit on older adults suicides and Severe Acute Respiratory Syndrome (SARS) epidemic in Hong Kong. Int J Geriatr Psychiatry. 2008;23(12):1231–8. 10.1002/gps.2056. 18500689 10.1002/gps.2056

[31] https://www.data.gov.qa/pages/dashboard-covid-19-cases-in-qatar/. Accessed 15 May 2021.

[32] https://www.psa.gov.qa/en/statistics1/Pages/Overview.aspx. Accessed 15 May 2021.

[33] https://www.moph.gov.qa/english/strategies/Supporting-Strategies-and-Frameworks/SummaryNationalMentalHealthFramework2019-2022/Pages/default.aspx. Accessed 15 May 2021.

[34] Wadoo O, Ahmed MAS, Reagu S, Al Abdulla SA, Al Abdulla MAY. Primary care mental health services in Qatar. BJPsych Int. 2021;18(1):15–8. 34287398 10.1192/bji.2020.45 PMC8274405

[35] Elzamzamy K, Alsiddiqi A, Khalil A, Elamin H, Karim MA, Wadoo O. Newspaper depiction of mental and physical health in Qatar. BJPsych Int. 2020. 10.1192/bji.2020.11. 10.1192/bji.2020.11 PMC8274433 34287419

[36] Rajkumar RP. COVID-19 and mental health: a review of the existing literature. Asian J Psychiatr. 2020;52: 102066. 32302935 10.1016/j.ajp.2020.102066 PMC7151415

[37] https://ourworldindata.org/policy-responses-covid. Accessed 15 May 2021.

[38] Franchini S, Spessot M, Landoni G, Piani C, Cappelletti C, Mariani F, Faccincani R. Stranger months: how SARS-CoV-2, fear of contagion, and lockdown measures impacted attendance and clinical activity during February and March 2020 at an urban Emergency Department in Milan. Disaster Med Public Health Prep. 2020. 10.1017/dmp.2020.265. 32713377 10.1017/dmp.2020.265 PMC7588723

[39] Jeffery MM, D’Onofrio G, Paek H, Platts-Mills TF, Soares WE, Hoppe JA, Melnick ER. Trends in emergency department visits and hospital admissions in health care systems in 5 states in the first months of the COVID-19 pandemic in the US. JAMA Intern Med. 2020;180(10):1328–33. 32744612 10.1001/jamainternmed.2020.3288 PMC7400214

[40] Bryan CJ, Bryan AO, Baker JC. Associations among state-level physical distancing measures and suicidal thoughts and behaviors among US adults during the early COVID-19 pandemic. Suicide Life Threat Behav. 2020;50(6):1223–9. 32589801 10.1111/sltb.12653 PMC7362130

[41] Killgore WD, Cloonan SA, Taylor EC, Allbright MC, Dailey NS. Trends in suicidal ideation over the first three months of COVID-19 lockdowns. Psychiatry Res. 2020;293: 113390. 32835926 10.1016/j.psychres.2020.113390 PMC7430225

[42] Wadoo O, Latoo J, Reagu SM, Amro RAA, Masoodi NA, Alabdulla M. Mental health during COVID-19 in Qatar. Gen Psychiatr. 2020;33(6): e100313. 10.1136/gpsych-2020-100313. 33195990 10.1136/gpsych-2020-100313 PMC7594533

[43] Karim MA, Wadoo O, Reagu SM, Amro R, Al Abdulla M. Telepsychiatry in the Arabian Gulf region-implications beyond the Covid-19 pandemic. Asian J Psychiatry. 2020. 10.1016/j.ajp.2020.102397. 10.1016/j.ajp.2020.102397 PMC7455557 33271697

