# Supplementary material for: Suicidal behavior in a migrant majority population and impact on trends during the early Covid-19 period: a cross sectional study in Qatar
Source: Discov Psychol. 2022 May 25;2(1):28. doi: 10.1007/s44202-022-00040-8 (PMC9130969; doi:10.1007/s44202-022-00040-8)
Supplement: Supplementary file 1 — (PDF 90 KB) [file 44202_2022_40_MOESM1_ESM.pdf]

The Incidence and Risk Factors of Suicidal Behavior following the Coronavirus Disease 2019

* Required

Demographic Variables

1. Who are you ? 😊
   Please Identify yourself! *

   - ◯ Dr. Rajeev Kumar
   - ◯ Dr. Nahid Fadul
   - ◯ Dr. Marwa ElZain
   - ◯ Dr. Sagda Kunna
   - ◯ Dr. Ibrahim
   - ◯ Dr. Mohamed Hassan

2. ID# *

   PLEASE, Make sure you don't duplicate ID number!

3. Date of visit to ED *

It is already stated in the Excel sheet, but worth checking too!

Please input date (M/d/yyyy)

4. Is it a case or not? *

Case = Suicidality and/or Self-Harm in general

◯ Yes

◯ No

5. Gender *

◯ Male

◯ Female

6. Age *

In Years

7. Employed? *

Tips: Look at "Patient Information" section, from Old EMR or early notes on CERNER BE CAREFUL! This information might be located else where in the chart - e.g. Admission note or Discharge Summary or Case-manger or Social-worker notes

◯ Yes

◯ No

◯ Not available

8. If employed, What was the patient's job?

Just copy-paste from CERNER

9. Marital status *

Tips: Look at "Patient Information" section, from Old EMR or early notes on CERNER BE CAREFUL! This information might be located else where in the chart - e.g. Admission note or Discharge Summary or Case-manger or Social-worker notes

- ○ Single
- ○ Married
- ○ Divorced
- ○ Separated
- ○ Widowed
- ○ Unknown/Not available

10. If married, how many children does the patient have?

	0	1	2	More than 2	N/A
Number of children	○	○	○	○	○

11. Living status *

Tips: Look at "Patient Information" section, from Old EMR or early notes on CERNER BE CAREFUL! This information might be located else where in the chart - e.g. Admission note or Discharge Summary or Case-manger or Social-worker notes

- ◯ Living alone
- ◯ With others in group
- ◯ Living with family
- ◯ Unknown/Not available

12. Religion *

Tips: Look at "Patient Information" section, from Old EMR or early notes on CERNER BE CAREFUL! This information might be located else where in the chart - e.g. Admission note or Discharge Summary or Case-manger or Social-worker notes

- ◯ Islam
- ◯ Christian
- ◯ Hindu
- ◯ Others
- ◯ Unknown/Not available

13. Residency *

Hint: Check social worker/case management notes Sometime, it might be easily inferred - e.g. statements like " ... has been working since 5 years in Qatar " or " ...came to Qatar few days ago looking for a job "

- ◯ Citizen
- ◯ Resident
- ◯ Visitor
- ◯ Others
- ◯ Unknown/Not available

14. Duration of Residence *

Numbers of years in Qatar If NOT AVAILABLE, Please just type " NA"

15. Travel Restriction due to COVID-19? *

At the time of presentation to ER, was travel restriction due to COVID19 a contributing stress factor? Hint: This applies more to later months of the study. YES = CLEARLY documented evidence that travel-restriction contributed

- ◯ Yes
- ◯ No
- ◯ Unknown/Not available

Clinical Variables

16. Current smoking? *

Tips: Look at "Patient Information" section, " History" section, from Old EMR or early notes on CERNER BE CAREFUL! This information might be located else where in the chart

- ◯ Yes
- ◯ No
- ◯ Unknown/Not available

17. Current alcohol intake? *

Tips: Look at "Patient Information" section, " History" section, from Old EMR or early notes on CERNER BE CAREFUL! This information might be located else where in the chart

- ◯ Yes
- ◯ No
- ◯ Unknown/Not available

18. Current substance use? *

Tips: Look at "Patient Information" section, " History" section, from Old EMR or early notes on CERNER BE CAREFUL! This information might be located else where in the chart

- ◯ Yes
- ◯ No
- ◯ Unknown/Not available

19. Past psychiatric history? *

- Yes
- No
- Unknown/Not available

20. Past psychiatric clear diagnosis *

Write exact diagnosis as in the File If NO past psychiatric history, please type " NA"

21. Known to our Mental Health Services? *

Was it the first (New) presentation to our Mental Health Services?

- Yes
- No

COVID-19-related variables

22. COVID-19 positive? *

Hint: Check 'Results View for the past 18 months'

- ◯ Yes
- ◯ No
- ◯ Unknown/Test not done

23. Any treatment received for COVID-19 during the study period? *

"Treated for COVID19" = Treatment directed at managing symptoms/distress related to COVID19 disease process - e.g. Severe respiratory distress with Chloroquine, Hydroxychloroquine etc. *Check medications lists!

- ◯ Yes
- ◯ No

24. Recovered from COVD-19? *

Had +ve COVID19 status then became -ve --> Check 'Results View for the past 18 months' Or Developed symptoms then resolved

- ◯ Yes
- ◯ No
- ◯ Unknown/Not available

25. Current psychiatric symptoms at presentation at index presentation *

- [ ] Aggression
- [ ] Anxiety
- [ ] Bizarre/Disorganized behavior
- [ ] Confusion/Delirium
- [ ] Depressive Symptoms
- [ ] Insomnia
- [ ] Psychosis - e.g. Hallucinatory behavior, suspiciousness, formal thought disorder etc.
- [ ] Wandering
- [ ] Other(s)

26. Current psychiatric diagnosis *

Write exact diagnosis as in the File

27. Is there any documentation on current hopelessness? *

Hint: Documented statements like ' Death wishes ', 'Feels hopeless', 'Desperate about situation', 'Life is worthless' etc. etc. YES = CLEARLY documented that the patient exhibited any of the above NO = CLEARLY documented that the patient DID NOT exhibit any of the above UNKNOWN/NOT AVAILABLE = No documentation suggestive of that

- ( ) Yes
- ( ) No
- ( ) Unknown/Not available

Suicide-related variables

28. Thoughts of suicide at presentation? *

YES = CLEARLY documented that the patient exhibited suicidal thoughts NO = CLEARLY documented that the patient DID NOT exhibit suicidal thoughts UNKNOWN/NOT AVAILABLE = No documentation suggestive of that

- Yes
- No
- Unknown/Not available

29. Thoughts of deliberate self-harm (but no intent to die) at presentation? *

YES = CLEARLY documented that the patient exhibited self-harming thoughts NO = CLEARLY documented that the patient DID NOT exhibit self-harming thoughts UNKNOWN/NOT AVAILABLE = No documentation suggestive of that

- Yes
- No
- Unknown/Not available

30. Attempted suicide? *

e.g. hanging, jumping, serious OD ***Action(s) with a clearly documented intent to end one's life **Be careful of accidental overdoses and other accidental acts! YES = CLEARLY documented that the patient attempted suicide NO = CLEARLY documented that the patient DID NOT attempt suicide - e.g. 'No suicidal attempts were made ' UNKNOWN/NOT AVAILABLE = No documentation suggestive of that

- Yes
- No
- Unknown/Not available

31. Attempted self-harm? *

e.g. superficial cutting, sma; OD etc. ***Action(s) with a clearly documented intent NOT to end one's life, rather for another reason **Be careful of accidental overdoses and other accidental acts! YES = CLEARLY documented that the patient attempted self-harm NO = CLEARLY documented that the patient DID NOT attempt self-harm - e.g. 'No self-harming attempts were made' UNKNOWN/NOT AVAILABLE = No documentation suggestive of that

- ○ Yes
- ○ No
- ○ Unknown/Not available

32. What was the suicidal or self-harming behavior documented on presentation? *

*You can choose as many options as you find applicable! ***If not suicidal or self-harming, please select "NOT_APPLICABLE" **Be careful of accidental overdoses and other accidental acts!

- ☐ Firearm
- ☐ Hanging
- ☐ Hitting self
- ☐ Jumping from height
- ☐ Jumping in front of running cars
- ☐ Overdosing
- ☐ Stabbing
- ☐ Slashing / Self-cutting and mutilation
- ☐ Self-injecting with medicine(s)/drug(s)
- ☐ Other(s)
- ☐ NOT_APPLICABLE

33. Past history of suicidal or self-harming behaviors or thought? *

Yes = CLEARLY documented that the patient had history of etc. etc. No = CLEARLY documented that the patient DID NOT have any history of etc. etc. Unknown/Not available = Not documented at all

	Yes	No	Unknown/Not available
Past history of suicidal thoughts (with no attempts)?	◯	◯	◯
Past history of suicidal attempts/behavior(s)?	◯	◯	◯
Past history of self-harming thoughts (with no attempts)?	◯	◯	◯
Past history of self-harming attempts/behavior(s)?	◯	◯	◯

34. Family history of self-harming or suicidal behavior(s)? *

◯ Yes

◯ No

◯ Unknown/Not available

Stress-related variables

35. Evidence of recent acute stressor(s) *

- ◯ Yes
- ◯ No
- ◯ Unknown/Not available

36. Give details of stressors below: *

e.g. loss of job, financial issues, domestic violence etc.). List simple in brief words

37. Select stressors contributing to the documented self-harming or suicidal behavior *

*You can choose as many options as you find applicable! *If there were no stressors, please choose ' NOT_APPLICABLE"

- ☐ COVID19 related issues (e.g. receiving +ve results, worries about family members being infected etc.)
- ☐ Deportation
- ☐ Family-related problems
- ☐ Financial difficulties
- ☐ Legal problems
- ☐ Physical Health issues (Not COVID19 related)
- ☐ Work-related problems
- ☐ Termination/Loss of job
- ☐ Other(s)
- ☐ NOT_APPLICABLE

38. Evidence of quarantine? *

Hint: More applicable to cases presented in recent months YES = Documented evidence that the patient was quarantined, transferred to COVID19 site etc. NO = CLEARLY documented evident that the patient did not need to be quarantined and stayed at a non-COVID19 site including his/her home UNKNOWN/NOT AVAILABLE =Unable to determine this occurrence based on documentation

- ◯ Yes
- ◯ No
- ◯ Unknown/Not available

39. Any additional information unique or relevant to the case or any problems encountered:

This content is neither created nor endorsed by Microsoft. The data you submit will be sent to the form owner.

Microsoft Forms
